# Fungal Biomarkers Stability in Mars Regolith Analogues after Simulated Space and Mars-like Conditions

**DOI:** 10.3390/jof7100859

**Published:** 2021-10-14

**Authors:** Alessia Cassaro, Claudia Pacelli, Mickael Baqué, Jean-Pierre Paul de Vera, Ute Böttger, Lorenzo Botta, Raffaele Saladino, Elke Rabbow, Silvano Onofri

**Affiliations:** 1 Department of Ecological and Biological Sciences, University of Tuscia, Largo Dell’Università snc, 01100 Viterbo, Italy; cassaro@unitus.it (A.C.); lorenzo.botta@unitus.it (L.B.); saladino@unitus.it (R.S.); onofri@unitus.it (S.O.); 2 Italian Space Agency, Via del Politecnico snc, 00133 Rome, Italy; 3 German Aerospace Center (DLR), Planetary Laboratories Department, Institute of Planetary Research, Ruthefordstraße 2, 12489 Berlin, Germany; mickael.Baque@dlr.de; 4 MUSC, German Aerospace Center (DLR), Space Operations and Astronaut Training, 51147 Köln, Germany; jean-pierre.devera@dlr.de; 5 German Aerospace Center (DLR), Institute of Optical Sensor Systems, 12489 Berlin, Germany; ute.Boettger@dlr.de; 6 Radiation Biology Division, Institute of Aerospace Medicine, DLR, Linder Höhe, 51147 Köln, Germany; elke.rabbow@dlr.de

**Keywords:** spectroscopy, Mars exploration, life-detection, pigments, nucleic acids

## Abstract

The discovery of life on other planets and moons in our solar system is one of the most important challenges of this era. The second ExoMars mission will look for traces of extant or extinct life on Mars. The instruments on board the rover will be able to reach samples with eventual biomarkers until 2 m of depth under the planet’s surface. This exploration capacity offers the best chance to detect biomarkers which would be mainly preserved compared to samples on the surface which are directly exposed to harmful environmental conditions. Starting with the studies of the endolithic meristematic black fungus *Cryomyces antarcticus*, which has proved its high resistance under extreme conditions, we analyzed the stability and the resistance of fungal biomarkers after exposure to simulated space and Mars-like conditions, with Raman and Gas Chromatography–Mass Spectrometry, two of the scientific payload instruments on board the rover.

## 1. Introduction

In the next few years, in situ space exploration missions will be devoted to the detection of biogenic signatures of extinct or extant life on Mars. The driver for searching for life on Mars is the findings supporting that ancient environments on Mars could have supported microbial life [1]. One of the primary issues in the search for life is that the present-day Martian surface presents a very inhospitable habitat for life as we know it because of the intense radiation, highly oxidizing conditions, concentrated evaporative salts, and extremely low water activity [2]. Despite the Martian surface having been cold and predominantly dry for at least the last three billion years (i.e., the Amazonian Period, immediately following the Hesperian), the subsurface could have sustained stable reservoirs of geothermally heated liquid water for the majority of this time. These conditions could represent a long-lived habitat that maintained hypothetical living cells [3,4,5,6]. For these reasons, the next planetary mission, ESA-Roscosmos ExoMars Rosalind Franklin, will collect and analyze samples in the subsurface, up to 2 m depth [7,8,9], to access places where organic molecules may be well preserved even after billions of years [10]. 

In this context, our best chance to find traces of extant or recently extinct life on Mars is to look for biomarkers [11]. A molecular biomarker is defined as a pattern, distribution of molecules or molecular structures that in nature derives uniquely from past or present biological processes [12,13]. Indeed, we consider as a possible biomarker a range of molecules indicative for life as we know it, such as DNA, amino acids, lipids, carbohydrates, pigments, intermediary metabolites [13], their degradation products and their general characteristics as preservation potential, specificity and extractability [14]. Assuming that hypothetical Martian life is similar to that on Earth, the identification of these compounds would be diagnostic of extant or recently extinct life because they are the indispensable biological components of the organisms as we know it. Any extinct or existing forms of microbial life on Mars may have produced biomolecules that may still be preserved and detectable in Martian rocks. In fact, minerals and organic molecules are strictly linked: minerals provide surfaces to support, concentrate, and preserve organic molecules. In support of this, organic matter was recently found within the three-billion-year-old mudstones of Gale Crater [15]. 

Due to the capability to detect biomolecules or their alteration products within mineral grains, Raman spectroscopy is part of the analytical instrumentation in the payloads of the rovers. This technique, on top of its main goal to provide mineralogical identification of the samples, can also detect a wide range of potential biomarkers in the rock substrate. It has been chosen for its non-destructive properties, for its sensibility during the detection of microbial life closed in their niches, and for its efficiency in in situ analyses also in presence of mineral rocks [16].

The Mars Organic Molecule Analyzer (MOMA), among various instruments, will operate with Pyrolysis–Gas Chromatography–Mass Spectrometry (Pyr-GCMS) in order to analyze volatile compounds in the Martian subsurface. In this context, it is important to understand the interaction effect of high temperatures with regolith and organic matter. In extreme environments, life could find refuge inside rocks, as in terrestrial analog environments, where in harsh conditions, extremophilic microorganisms dwelling inside the subsurface produce characteristic compounds protecting themselves from stressed conditions [17,18], among which are carotenoids and melanin pigments. Organic, metabolic and morphological structure or compounds of the organisms could persist in mineral structures and are considered good biomarkers of microorganisms’ presence. On Earth, biological matter can be preserved in sedimentary rocks as carbonaceous macromolecules [12] and maybe, if life exists or existed on Mars, its rocks could preserve organic deposits: organic components may be preserved either as degraded molecules within mineral structures or as disseminated molecules chemically bonded to mineral particles of rocks, such as phyllosilicates [19]. 

In this context, the BIOMEX (BIOlogy and Mars EXperiment) project, aimed at investigating the endurance of extremophiles and the stability of biomolecules under space and Mars-like conditions in the presence of Martian regolith analogs [20]. This experiment involved 16 months of real space and a close approach to Mars-like conditions exposure outside the International Space Station (ISS). The experiment was placed externally aboard the EXPOSE-R2 exposure payload and comprised a series of ground-based simulation tests, including Experiment Verification Test (EVT) and Science Verification Tests (SVTs), carried out before the flight. In the frame of ground-based tests, the stability/degradation of biomolecules of the extremotolerant microorganism *Cryomyces antarcticus* was investigated; the fungus, isolated from the McMurdo Dry Valleys (South Victoria Land, Antarctica), was chosen for its widely proved ability to withstand stressors similar to the ones encountered in space and Mars-like environments (e.g., ionizing and no ionizing radiation, vacuum or Martian atmosphere, temperature cycles) [21,22].

In total, fungal colony samples were investigated after exposure to simulated space and Mars-like conditions during the ground-based experiments before flight. Fungal colonies were spread on three different cultivation media consisting of Malt Extract Agar (MEA) and three different regoliths: the Original Substrate (i.e., Antarctic sandstone, OS), the Phyllosilicatic Mars Regolith Simulant (P-MRS) analogue and the Sulfatic Mars Regolith Simulant (S-MRS) analogue. The P-MRS simulates igneous rocks altered by hydrous fluids (neutral to basic) while the S-MRS mimics a more acidic environment with sulfate deposits [23]. The objectives of this study were to identify potential fungal biomolecules to be accounted as biomarkers and to understand if and how simulated space and Mars environment could modify them. As the primary goal of this study was the detection of microbial signatures within the Martian regolith analogues, the same methods, Raman spectroscopy and Gas Chromatography–Mass Spectrometry (GC-MS), planned for the ExoMars Rosalind Franklin mission, were used. In addition, extracted melanin pigments and nucleic acids were analyzed by using UV–VIS spectrophotometry and by quantitative Polymerase Chain Reaction (qPCR) technique, respectively, to detect any changes in structure after simulated space and Mars exposure.

## 2. Materials and Methods

### 2.1. Ground-Based Simulations 

#### 2.1.1. Science Verification Tests (SVTs)

The SVTs were performed at the Planetary and Space Simulation facilities (PSI) at the Institute of Aerospace Medicine (German Aerospace Center, DLR, Koln, Germany). SVTs are designed to ensure that all samples are appropriately prepared to successfully withstand hardware integration, conditions experienced during the mission, and post flight de-integration. The application of mission-equivalent space parameters allows for testing the resilience of samples toward the extreme environmental conditions of space and simulated Mars exposure on the ISS. Samples were accommodated in wells with a diameter of 12 mm, within square aluminum alloy carriers, with a side of 76 mm. Following the accommodation plan scheduled for the EXPOSE-R2 mission, SVTs allowed only one replicate per sample [20]. To simulate space-like test conditions, the sample that was grown on OS analogue was exposed to vacuum (10^−5^ Pa) and cycling temperatures between −25 °C (16 h in the dark) and +10 °C (8 h during irradiation), alone (Bottom samples) or in combination with polychromatic UV (200–400 nm) radiation produced by the solar simulator SOL2000 (Top samples). The dose of 570 MJ/m^2^ was reached by running the solar simulator (SOL2000; Dr. Hönle GmbH, Germany, Rabbow et al., 2017) for 125 h at 1271.2 W/m^2^. In parallel, Mars test parameters were simulated by low temperature (−25 °C), Mars-like atmosphere (95.55% CO_2_, 2.70% N_2_, 1.60% Ar, 0.15% O_2_, *370 ppm H_2_O; Praxair Deutschland GmbH), and Mars-like pressure of 10^3^ Pa for S-MRS and P-MRS analogues, alone or in combination (Top samples) with the same radiation as described above. Neutral density filters (0.1%) were used to attenuate radiation in all tests performed; all conditions were simulated for a period of 28 days. The applied fluency corresponds to the long-term space experiment of 1 year of exposure outside the ISS, as estimated from previous EXPOSE data and simulations [24,25]. It should be noted that space parameters cannot be fully mimicked in the laboratory (space vacuum and complex radiation environment), for example, deep UV, that is, solar UV radiation below 200 nm. Below the irradiated samples, an identical set of samples (space dark samples and Mars dark samples/bottom samples) was kept in the dark and experienced all simulation parameters except UV radiation exposure. Controls (Ctr) were kept at DLR in the dark at room temperature. The exposure conditions are summarized in Table 1.

#### 2.1.2. Fungal Melanin Extraction

Melanin was extracted from dried colonies of *C. antarcticus* grown on different substrata, optimizing the protocol reported by [26]. Fungal cells were collected by centrifugation at 16,100 rcf for 20 min, washed with phosphate-buffered saline (PBS) (pH 7.4). Cells were suspended in 20 µL of a buffer composed by 0.25 µL of enzyme from *Trichoderma harzarium* (Sigma #L1412, St. Louis, MO, USA), 50 µL of 0.2 M sodium citrate (pH 5.5), 15 µL of 0.2 M citric acid and 18.2 g of sorbitol, up to the final volume of 500 µL.

Samples were incubated overnight at 30 °C, shaken at 50 rpm, washed with PBS two times and collected by centrifugation (10 min at 16,100 rcf). The supernatant was removed by adding 1 mL of 4 M guanidine thiocyanate (VWR International srl, Radnor, PA, USA), and samples were incubated overnight on stirrer. Samples were washed with PBS and centrifuged for 15 min at 16,100 rcf for two times. Then, 1 mg/mL of Proteinase K, previously dissolved in the reaction buffer (4 µL of TRIS HCl, 1 µL of CaCl_2_ and 1 mL of SDS, up the final volume of 20 mL) was added to the samples; they were incubated at 37 °C for 4 h and after centrifuged for 5 min at 16,100 rcf. Samples were washed two times by adding 1 mL of PBS 4×, and three times by adding 1 mL of chloroform.

A volume of 2 mL of a 6 M HCl solution was added to the samples, and they were boiled for 1 h. Samples were transferred in dialysis membrane (SnakeSkin Dialysis Tubing, 3.5 K MWCO-Thermo Scientific, Waltham, MA, USA) in sterile water for 3 days, changing the water every day. Finally, samples were lyophilized overnight with the Lyophilizer FreeZone 2.5 L (Freeze Dry Systems, LabConco, Kansas city, MO, USA) and then used for the analyses. 

#### 2.1.3. Spectrophotometric Analysis

After the extraction, purified pigments were dissolved in 500 µL of NaOH 1 M and its UV-Visible spectrum was measured in a UV spectrophotometer (UV 1600 PC Spectrophotometer, VWR International) by using M.Wave professional 2.0. A standard graph for estimation was used and was made using synthetic melanin. NaOH 1 M was used as a blank and the instrument was set in a range of 200–800 nm for the analysis. The correlation between absorbance and wavelength was defined. To determine the concentration of extracted melanin, synthetic DHN (1,8-DiHydroxyNaphthalene) melanin (Thermo-Fisher Scientific, Waltham, MA, USA) was prepared in 1 M NaOH at concentrations of 500 mg/mL and as reported in [27], a standard curve at 650 nm was obtained.

#### 2.1.4. Confocal Raman Spectroscopy Analyses 

Raman spectroscopy was performed at German Aerospace Center in Berlin, using a 532 nm excitation laser, with a Confocal Raman microscope (WITec alpha300), at room temperature, under ambient atmospheric conditions. The spectral resolution of the spectrometer is 4–5 cm^−1^. Before the analyses, the spectrometer was calibrated with pure silicon and paracetamol test samples. A 10× Nikon objective, with a 0.25 numerical aperture, was used to focus the laser on a 1.5 μm spot. For single spectra, all measurements were performed at 0.1 mW laser power with 10 s integration and 50 accumulations. Image scans were done at 0.7 mW with 1 s and 1 accumulation (to avoid signal saturation or damaging effects) on three distinct areas up to 100 μm × 100 μm and up to 500 image points, thus collecting a minimum of 1000 measurements per sample.

All data analyses were performed with the WITec Project FIVE software. Parameters used for analysis were the value of signal coverage on random zones on the samples, to show the presence/absence of the signal defined as spectra having Signal to Noise Ratio (SNR) values superior to 5 and the value of SNR. To analyze the presence of the signal, the region of interest between 200 and 2000 cm^−1^ was cropped, then a fifth-order polynomial function was applied for background subtraction; and finally, SNR masks were applied. As previously described in [28] for carotenoid pigments, the SNR was defined as the height of the 1600 cm^−1^ peak divided by the noise represented by the standard deviation of a spectral region without features (1750 to 1950 cm^−1^). The position of the 1600 cm^−1^ peak was also derived from masks available in the WITec Project FIVE software.

### 2.2. Nucleic Acid Analysis

#### 2.2.1. Nucleic Acid Extractions from Synthetic Mars and Terrestrial Soils

DNA was extracted from colonies, using the Nucleospin Plant kit (Macherey-Nagel, Düren, Germany) following the protocol optimized for black fungi as reported in [29]. Before amplification, DNA was quantified using QUBIT system and diluted at the concentration of 0.1 ng/μL for the following analyses.

#### 2.2.2. Acid Nucleic Detection through Quantitative Real-Time PCR (qPCR)

Three different target genes were amplified with qPCR approach to investigate the differences in acid nucleic detection: a long-repeated fragment (Large Sub-Units, LSU gene of 939 bp), a short-repeated fragment of the same gene (LSU gene of 330 bp) and a small but non-repeated fragment in the genome (β-actin gene of 330 bp). qPCR was performed with a BioRad CFX96 real time PCR detection system (BioRad, Hercules, CA, USA) using primers targeting the fungal LSU rRNA gene and the β-actin gene: LR0R (ACCCGCTGAACTTAAGC, [30]) and LR5 (TCCTGAGGGAAACTTC, [31]), and ACT512-F (ATGTGCAAGGCCGGTTTCGC3) and ACT783-R (TACGAGTCCTTCTGGCCCAT) [32], respectively, each at 5 pmol final concentration. 

The primers LR0R-LR5 and LR0R-LR3 were used to amplify a 939 bp and 300 bp products, respectively, spanning the LSU region of rRNA encoding genes. The standard qPCR cycling protocol for both products, consisting of a denaturation step at 94 °C for 5 min, followed by 35 cycles of denaturing at 94 °C for 45 s, annealing at 52 °C for 30 s, and elongation at 72 °C for 2 min, was performed. The primers ACT512-F and ACT783-R are used to amplify a 330 bp product spanning the β-actin gene. The standard qPCR cycling protocol, consisting of a denaturation step at 95 °C for 10 min, followed by 35 cycles of denaturation at 95 °C for 15 s, annealing at 61 °C for 20 s, and elongation at 72 °C for 15 s, was performed. Fluorescence measurements were recorded at the end of each annealing step. After 35 cycles, a melt curve analysis was performed by recording changes in fluorescence as a function of raising the temperature from 60–90 °C in 0.5 °C per increments. All tests were performed in triplicate. 

#### 2.2.3. Statistical Analyses

For multiple data points, mean and standard deviation were calculated. Statistical analyses were performed by one-way analysis of variance (Anova) and pair wise multiple comparison procedure (*t* test), carried out using the statistical software SigmaStat 2.0 (Jandel Scientific, San Jose, CA, USA).

#### 2.2.4. Organic Compounds Detection by Gas Chromatography–Mass Spectrometry

Gas chromatography associated to mass spectroscopy has been selected for the analysis of samples onboard of the rover of the ESA-ExoMars mission [33]. Each sample (pellet) was grinded in agate mortar and then suspended in 2 mL of ethyl acetate. The mixture was left 4 h under magnetic stirring at room temperature. After this time, the suspension was filtered to remove the solid, and the solution obtained was concentrated under reduced pressure. After the extraction and fractionation of the samples with *N*,*N*-bis-trimethylsilyl trifluoroacetamide in pyridine (620 µL) at 60 °C for 4 h in the presence of betulinic acid [3β -hydroxy-20(29)-lupaene-oic acid] as the internal standard (0.2 mg), the Gas Chromatography–Mass Spectrometry was performed. Mass spectrometry was carried out through the following program: injection temperature 280 °C, detector temperature 280 °C, gradient 100 °C for 2 min and 10 °C for 60 min. To identify the structure of the products, two strategies were followed. First, the spectra were compared with commercially available electron mass spectra libraries such as NIST (Fison, Manchester, UK). Second, GC-MS analysis was repeated with standard compounds. All products have been recognized with a similarity index (SI) greater than 98% compared with that of the reference standards. The analysis was limited to products of ≥1 ng/mL quantity, and the yield was calculated as micrograms of isolated product. 

## 3. Results

### 3.1. Detection of Pigments by Spectrophotometric Analyses

Spectrophotometric analyses were performed on fungal melanin extracted from *C. antarcticus* colonies after exposure to simulated space conditions (OS) and Mars-like conditions (P-MRS and S-MRS). The wavelength of maximum absorbance was scanned at a range of 200 to 800 nm. The UV-visible absorbance spectrum of the purified pigments showed a strong absorbance in the UV region, and a characteristic absorption peak was observed at 230 nm corresponding to typical melanin UV absorption. The strong absorption in the UV region with a progressive decrease at high wavelength is due to the presence of complex conjugated structures in the melanin molecule [34,35]. The decrease in the absorption with increasing wavelength is linear in the case of melanin. 

**Figure 1:**
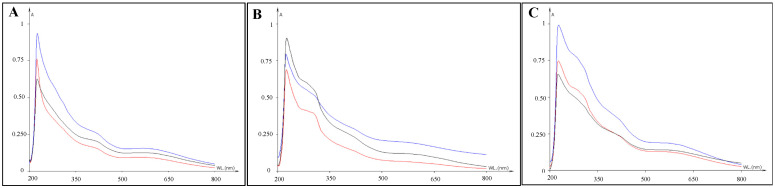
UV–VIS-spectra of melanin pigment extracted from *C. antarcticus* colonies grown on (**A**) OS exposed to simulated space conditions; (**B**) P-MRS and (**C**) S-MRS exposed to Mars-like conditions on different layers: Top (exposed to sun light with 0.1% Neutral Density filters) = red spectrum, Bottom (dark control in space, not exposed to space radiation) = blue spectrum and CTR (sample kept in the lab, in the dark at room temperature) = black spectrum. OS = Original Substrate, P-MRS = Phyllosilicatic Mars Regolith Simulant, S-MRS = Sulfatic Mars Regolith Simulant.

Figure 1 shows the melanin spectra extracted from samples exposed to simulated space UV radiation (OS Top; red line), vacuum (OS Bottom; blue line), to Mars-like UV radiation (P-MRS and S-MRS Top; red line) and Mars-like atmosphere (P-MRS and S-MRS Bottom; blue line) compared with relative control (black line). No change in melanin absorbance at 230 nm has been reported in all the experimental conditions (Top, Bottom and Control), compared with those reported in [36] for extracted melanin pigments from *C. antarcticus*. In addition, UV–VIS analyses of melanin pigments extracted for P-MRS and S-MRS analogues revealed a bulge at ~300 nm, not shown in OS spectra, probably due to the presence of regolith during the detection process. 

### 3.2. Detection of Pigments by Confocal Raman Spectroscopy

**Figure 2:**
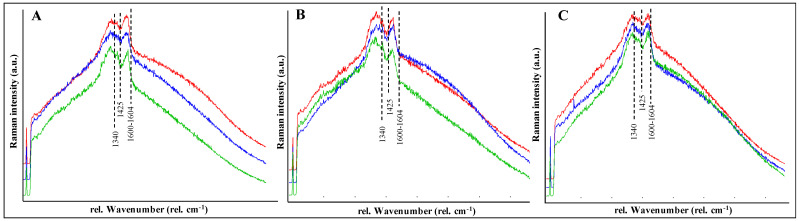
Raman spectra of melanin pigments of *C. antarcticus* grown on (**A**) OS exposed to space simulated conditions; (**B**) P-MRS and (**C**) S-MRS exposed Mars-like conditions on different layers: Top (exposed to sun light with 0.1% Neutral Density filters) = red spectrum, Bottom (dark control in space, not exposed to space radiation) = blue spectrum and Control (sample kept in the lab, in the dark at room temperature) = green spectrum. OS = Original Substrate, P-MRS = Phyllosilicatic Mars Regolith Simulant, S-MRS = Sulfatic Mars Regolith Simulant.

Colonies of *C. antarcticus* grown on different analogues were analyzed by Confocal Raman spectroscopy after exposure to simulated space and Mars-like conditions. The results of Raman analyses identified the melanin spectra with two main peaks, according to [37]: an intense and broad peak at 1590–1605 cm^−1^ and a second peak at lower wavenumber at 1340 cm^−1^ (Figure 2). The presence of these peaks is probably due to the aromatic C-N bonds for the first peak [38] and to the stretching of the C-C bonds within the rings of the aromatic melanin monomers for the second peak [39]. The presence of other peaks and shoulders in all spectra, with the most prominent one around 1425 cm^−1^, served as a proof that the samples did not undergo thermal degradation and that the signal acquired is of melanin and not burnt organic matter (or amorphous carbon). Figure S1 shows the signal coverage for the image scan analyses, calculated from the application of a SNR superior to 5; results were normalized to the corresponding SNR of the non-irradiated sample (Ctr) for each analogue.

No significant melanin changes are reported in all the collected spectra; similar peak positions (1602–1604 cm^−1^) were detected in samples exposed to simulated space conditions (OS) and in samples exposed to Mars-like conditions (P-MRS and S-MRS), both in Bottom and Top conditions, in comparison with the respective Control samples (Figure S2A,B).

### 3.3. Detection of Nucleic Acids through Amplification Method

Although a thermocycler instrument is not one of the pieces of equipment foreseen for the imminent exploration missions to Mars, it is a good candidate instrument to search for Earth-like life beyond Earth [40]. It is specific and sensitive, detecting even a single DNA molecule in a sample. The persistence of intact DNA has been tested on samples after a ground-based experiment simulating 16 months of exposure to space and Mars-like conditions outside the ISS. First, two different gene lengths of 939 bp and 330 bp, spanning the ribosomal LSU, have been targeted to be quantitatively amplified by qPCR based on the principle that damaged DNA is not amplifiable. Then, since the ribosomal genes occur in multiple copies in the genomes, we decided to amplify a region of 330 bp of the housekeeping gene β-actin, which is present in a single copy in the genome, to compare the level of detection based on the different gene length and number of copies in the genome.

**Figure 3:**
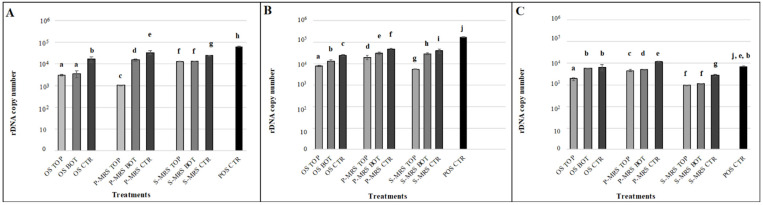
(**A**) Quantitative PCR (qPCR) of a 939 bp target LSU (Large SubUnit) gene; (**B**) a 330 bp target gene (LSU) and (**C**) a 330 bp target gene (actin) of *C. antarcticus* DNA, after exposure to SVT treatments. On the axis of the ordinates the number of amplified copies on a logarithmic scale is shown; on the abscissa axis, the treatments are as follows: DNA from samples exposed to space simulated conditions (OS) and samples exposed to Mars-like conditions (P-MRS and S-MRS). OS = Original Substrate (sandstone); P-MRS = Phyllosilicatic Mars Regolith Stimulant; S-MRS = Sulfatic Mars Regolith Stimulant. Top (exposed to sun light with 0.1% Neutral Density filters), Bottom (dark control in space, not exposed to space radiation) and CTR (sample kept in the lab, in the dark at room temperature); POS CTR = DNA of *C. antarcticus* colony growth in physiological conditions (cultivated on MEA and incubated at 15 °C). The same letters above the bars indicate that the values are not statistically significant according to the *t* test (*p* ≤ 0.05). OS = Original Substrate, P-MRS = Phyllosilicatic Mars Regolith Simulant, S-MRS = Sulfatic Mars Regolith Simulant.

In Figure 3, we reported the amplification of nucleic acids amplifying 939 bp and 330 bp of LSU gene and 330 bp of β-actin; the results showed a high amount of amplified DNA in all the experimental conditions tested and despite the gene type amplified: 13,170 DNA copies on average and never less than 10^2^ copies were amplified. The test highlighted a common trend for all the samples, i.e., a lower amount of amplified DNA for samples exposed to UV radiation and vacuum (Top), and a higher copy number for samples exposed to vacuum but no radiation (Bottom) and in the control samples. 

To conclude, all the gene amplifications worked out, and the number of amplified DNA copies was never under the amplification limit (one copy of a target sequence in genomic DNA), even when we decided to use the single copy gene β-actin.

### 3.4. Organic Compounds Detection by Gas Chromatography Associated to Mass Spectrometry

The Mars Organic Molecule Analyzer instrument onboard the ExoMars rover will employ thermal extraction protocols [41]. The characterization of thermal extracts will be carried out by GC-MS detection [42]. This analysis was performed only on a selection of samples, control and samples exposed to simulated space and Mars-like conditions (Top condition). 

The GC-MS analysis revealed the presence of several compounds using mass-to-charge ratios (*m/z*): azelaic acid (1,7-epta-didecanoic acid), myristic acid (1-tetradecanoic acid), pyruvic acid (2-Oxopropanoic acid), glucose, fructose, glucitol, glycerol (1,2,3-tri-hydroxy propane) and ethylene glycol (1,2-dihydroxy ethane). The identified compounds are shown in Table 2; the *m/z* fragmentation spectra of the identified compounds are reported in Table 3. Particularly, the results of pyrolysis analyses showed the presence of low-molecular-weight saturated carboxylic acids. Dicarboxylic acids, such as azelaic acid, were detected in Control and Top samples in OS, P-MRS, and S-MRS analogues (Table 2). In particular, azelaic acid was found in all examined samples, allowing to consider this molecule as a distinctive biomarker for the presence of *C. antarcticus*. Myristic acid was detected only in S-MRS samples exposed to Top conditions and in the relative Control (Table 2). Surprisingly, the amount of azelaic and myristic acids increased after the treatments. A low amount of pyruvic acid was found only in S-MRS control samples; no amount was shown in exposed samples, probably due to the effect of radiation. These results are similar to those reported for ethylene glycol, detected only in the case of S-MRS and P-MRS Top samples. 

## 4. Discussion

The detection of biomolecular markers in the subsurface regolith of Mars is a primary goal for astrobiology. Investigation on the stability or degradation-rate of any biomarker after exposure to simulated or real space conditions is of high interest for space exploration. The stability of any biomarker is dependent on its initial form, the matrix in which it is hosted, and the chemical and physical processes to which it is subjected over time [19].

In this paper, we aimed at investigating the biomarkers associated with fungal colonies in Martian regolith analogues under simulated space and Mars-like conditions, tested during the Scientific Verification Test of the BIOMEX project, with the same techniques planned for the robotic exploration of Mars. Assuming that life on Mars evolved with the same characteristics than that on Earth or that there was an exchange of material inside meteorites between Earth and Mars [43], it is reasonable to search for traces of Earth-like life on Mars. Among biomarkers, we first focused on pigments. The black fungus *C. antarcticus* can withstand the hyper-arid and extremely cold conditions of McMurdo Dry Valleys in Antarctica, thanks to its strongly melanized cell wall that helps to protect the fungus from the environmental stressors of these remote areas. It is known that the fungus is able to survive in different stressor conditions, such as ionizing radiation [36], heavy ions in hydrated and de-hydrated conditions [44,45]. 

Melanins are known to be involved in cellular resistance against a multitude of factors, such as toxic metals, hyperosmotic conditions and pH variations [46], but also extreme temperatures, desiccation and radiation, such as ultraviolet (UV) light, oxidizing agents and even ionizing radiation [35]. We aimed at detecting the presence of melanin pigments in samples subjected to simulated space and Mars-like exposure with different methods. Spectral analyses were used to confirm the presence and the possible alteration of melanin pigments after treatments. The UV spectrum (Figure 1) was characterized by the unaltered typical absorption profile of *C. antarcticus* melanin pigments [36]. It shows a strong absorption in the UV region (230 nm) with a progressive reduction as the wavelength increased, due to the presence of many complex conjugated structures in the melanin molecule [34,35]. This kind of analyses is relevant for the upcoming NASA Mars 2020 exploration rover Perseverance, which has onboard the SHERLOC (Scanning Habitable Environments with Raman and Luminescence for Organics and Chemicals) instrument, a deep UV laser with emission wavelengths below 250 nm that will provide an integrative analysis to avoid the fluorescence background during Raman measurements [47]. The importance of using a multiple technique approach to characterize melanic pigments is due to its absorption wavelength, which is similar to other compounds (e.g., amorphous carbon), making its detection more challenging. 

Indeed, to further investigate the state of preservation and characterize the fungal melanin, we performed Raman spectroscopy analysis. The Raman spectra were dominated by two main peaks around 1340 cm^−1^ and 1600 cm^−1^ and one smaller at 1425 cm^−1^ (Figure 2). According to the literature, the presence of the main peak at 1600 cm^−1^ is due to the stretching vibration of the aromatic C = C stretching modes and the 1340 cm^−1^ peak to the C-N stretching band of indole [48]: these peaks together with the smaller peak at 1425 cm^−1^ are attributed to the presence of melanin pigments and, hence, to the fungal colonies. 

It is well known that melanin pigments contribute to protecting microorganisms against the effect of radiation, which are abundant in space. Melanin pigments may have helped potential Earth-like life to survive in the harsh and highly irradiated environment of Mars, as it occurs in some extreme environments on Earth, where microorganisms have adopted this survival strategy. This assumption is supported by previous studies on the black fungus *C. antarcticus*; the melanized strain survived better the exposure to increasing doses of ionizing radiation compared to the non-melanized counterpart [49], confirming the protective role of melanin against ionizing radiation. 

Furthermore, the ExoMars rover is equipped with a pyrolysis gas chromatography–mass spectrometry device with the aim of detecting organic molecules such as carboxylic acids in Martian subsurface materials [35,41,42,43,44,45,46,47,48,49,50,51,52]. Assessing the possible thermal destruction and transformation of organic matter induced by pyrolysis is of utmost importance, for the next rover missions. Indeed, pyrolysis, may alter and transform organic matter [53,54], hiding the presence of organic signatures from samples. For example, organosulfur compounds that were detected by the Sample Analysis at Mars (SAM) instrument onboard the Curiosity rover may derive from artificial secondary reactions of sulfur (from decomposition of sulfates) with organic radicals [15], due to the high temperature of the pyrolysis processes. In this context, the main question is: do potential fungal biomarkers remain unaltered during pyrolysis processes? Our study reports that pyrolysis GC-MS is able to detect fungal signals, even when the biomarkers are present in low amounts. Indeed, our data highlighted the presence of low molecular weight carboxylic acids, such as pyruvic acid (only in S-MRS Control samples) and myristic acid (only in S-MRS Top and Control samples). Although pyruvic acid is a key intermediate in several metabolic pathways throughout the cell, it cannot be accounted as a biomarker since it has been found in carbonaceous meteorites, and it can be produced through the chemistry of interstellar nitriles, HCN/CN and ketene [55]. Conversely, myristic acid, which can be part of the phospholipid bilayer of the plasma membrane of the eukaryotic cell, can be considered as biomarker. Nevertheless, in our study, it has been found only in Top and Control S-MRS samples. Interestingly, azelaic acid, which is a saturated dicarboxylic acid involved in stress resistance, was found in all tested samples, without any alteration in GC-MS profiles (Table 2). In this framework, we can define this dicarboxylic acid directly related to the presence of fungal colonies, and we can define it as a good biomarker for the search of Earth-like life elsewhere. Although they can be generated by both biotic and abiotic synthesis (Fischer–Tropsch reactions), in the latter, carbons are added one at a time. In most microorganisms, fatty acids are generated biochemically by the addition of two carbon atoms at a time [56]. This can help discriminating biotic and abiotic origins of the molecules, and therefore, it allows to consider fatty acids with an odd C number as a potential biomarker. The other detected compounds in our experiment, such as carbohydrates components (glucose and fructose) may derive from the malt extract used for the cultivation of *C. antarcticus*. The low amount of ethylene glycol detected only in S-MRS and P-MRS irradiated samples most probably derived from the degradation of more complex substances. Even if we can correlate the presence of the colony with the detection of azelaic acid through the GC-MS analyses, the compound was detected in a very small amount; this has to be taken into account in future space exploration missions, in order to avoid negative outcomes. One of the restrictions of this technique is the undetectability of high molecular weight compounds. Indeed, one of the main features of the black fungus, the melanin pigments, was not detected using GC-MS instrumentation, showing the importance of using multiple and connected techniques to avoid false negative results.

Then, we investigated the possibility to detect fungal DNA after the exposure through quantitative PCR. Nucleic acids are an ideal target biomarker due to their unambiguity, non-specificity and mainly the impossibility of generation in the absence of life [57,58]. DNA potentially preserved in Martian soil may be destroyed by the effects of UV, cosmic ray exposure and radioactive decay on nucleic acids [59,60]. Ancient DNA is prone to damage (e.g., hydrolysis, depurination) and fragmentation [9,61] once it is no longer actively repaired in a biological system. However, as previously reported in [62] and in [63], DNA showed to be still detectable in environmental samples by PCR and stable in Mars-like conditions [64]. In addition, the bound between DNA and minerals may have facilitated the preservation on different geological timescales, even more in cold and dry environments, such as the Gale Crater on Mars with an average temperature of −48 °C [65]. In the framework of planetary exploration, the implementation of techniques, such as the qPCR that allows to amplify fragments of a DNA molecule, would detect even minimal traces of DNA, even after partial degradation. Such kind of instruments are also very easy to miniaturize. It is already used in field campaigns with the MinION device [66]. This miniaturized technology was used for the first time in 2016 in the frame of Biomolecule Sequencer project [67,68] with the aim to detect unambiguous signs of life through nucleic acids sequencing, starting from a small amount of sample. The advantage of the in situ DNA detection with a miniaturized tool also allows minimizing any eventual contamination from terrestrial microorganisms [69]. Accordingly, our results suggested that, in spite of the treatments, the nucleic acids are still detectable by qPCR technique. In particular, we found no differences in the amplified region: an average of ~13,000 DNA copy number were detected. The advantage of the amplification of long-repeated LSU gene allows detecting any modification at the genomic level in an extended region. On the other hand, these results may give a false outcome, disguising possible radiation damage on DNA, due to the repetition of the gene in the whole genome. Indeed, the choice of β-actin gene amplification permits to amplify a small non-repeated gene region, with the objective to identify nucleic acid damage in a single copy gene. Therefore, we recommend that space agencies should investigate the utility of establishing and promoting a miniaturized PCR instrument (e.g., MinION) to promote the detection of nucleic acids beyond Earth. Specific primers should be used for each of the three domains: bacteria, archaea, and eukarya. In order to identify microorganisms by their sequence, an automated sequencer needs be included in addition to the thermocycler apparatus. Our results showed that, even when using instrumentation already planned for future missions, each analytical technique has constraints that limit the set of information produced. Further improvements in technique and instrumentation, as well as cross comparisons in varying approaches, will be required to optimize data interpretation for the upcoming missions to Mars.

## 5. Conclusions

The final aim of this work was to find a good approach to detect fungal biomarkers and to evaluate their detectability after exposure to simulated space and Mars-like conditions as well as to test instrumentation applicability for in situ analyses. Additionally, this work is useful to outline a database of biomarker of Earth microbial life, to improve the detection of biomarkers at Mars and to eliminate false-positives or negative detections [70]. In this context, we may consider melanin pigments as a fungal biomarker, owing to their high stability when detected in Martian regolith analogues after simulated space and Mars-like exposure. In addition, the detection of fatty acids through a GC-MS approach is of utmost importance since these molecules are found in the membranes of all living organisms and then, considered as potential biomarkers for life. Finally, although nucleic acids represent a controversial issue in the context of biomarkers, due to their lower stability and preservation over the time, our results demonstrate a good amplification and stability after the exposure to simulated space and Martian conditions. The results of this work are very appropriate given that the ExoMars Rosalind Franklin rover includes within its payload a Raman Laser Spectrometer and a Gas Chromatography–Mass Spectrometry instrument. 

## Figures and Tables

**Table 1:** Exposure conditions during the Scientific Verification Tests (SVTs).

Test Parameters	Duration
Vacuum (2 × 10^−4^) + polychromatic UV irradiation (200–400 nm), with SOL2000 at 1271.2 W/m^2^, attenuated with 0.1% neutral density filter.	28 days
	SOL2000
	125 h
Simulated CO_2_ Mars atmosphere 10^3^ Pa+ polychromatic UV irradiation (200–400 nm), with SOL2000 at 1271.2 W/m^2^, attenuated with 0.1% neutral density filter.	28 days
	SOL2000
	125 h
Control experiment, 1 atm air, dark, room temperature	28 days

SOL = Solar simulator.

**Table 2:** Abundance of main compounds detected with Gas Chromatography–Mass Spectrometry analysis after ground-based simulation.

Compounds ^[a]^	OS Top	OS Control	P-MRS Top	P-MRS Control	S-MRS Top	S-MRS Control
Azelaic acid	0.99	0.13	0.69	0.45	0.30	0.02
Myristic acid	nd	nd	nd	nd	0.48	0.42
Pyruvic acid	nd	nd	nd	nd	nd	0.17
Glucose	3.14	2.78	nd	nd	0.68	13.87
Fructose	0.64	7.53	nd	nd	nd	4.50
Glucitol	nd	3.58	nd	nd	0.88	2.50
Glycerol	nd	nd	nd	nd	nd	nd
Ethylene glycol	nd	nd	1.73	nd	0.41	nd

[a] The analyses were performed after silylation with *N*,*N*-bis-trimethylsilyl trifluoroacetamide in pyridine (620 µL) at 60 °C for 4 h in the presence of betulinic acid [3b-hydroxy-20(29)-lupaene-oic acid] as the internal standard (0.2 mg). All quantities are expressed in μg. GC-MS analysis of OS, P-MRS, and S-MRS samples. Original substrate (OS), Phyllosilicatic Mars Regolith Simulant (P-MRS) and the Sulfatic Mars Regolith Simulant Phyllosilicate (S-MRS). Top samples exposed on the Top of the payload in comparison with Control samples, not exposed to treatments. nd: not determined.

**Table 3:** Mass-to-charge ratio (*m/z*) value and the abundance of peaks of identified compounds.

Products ^[a]^	*m/z* (%)
Azelaic Acid ^[c]^	317 (25) [M-CH_3_], 302 (3) [M-2xCH_3_], 243 (2) [M-OSi(CH_3_)_3_], 201 (15) [M-Si(CH_3_)_3_-CO_2_-CH_3_], 186 (3) [M-2xSi(CH_3_)_3_], 170 (4) [M-OSi(CH_3_)_3_- Si(CH_3_)_3_], 73 (100).
Myristic Acid ^[b]^	300 (10) [M], 285 (95) [M-CH_3_], 257 (3) [M-2xCH_3_], 73 (100).
Palmitic Acid ^[b]^	328 (20) [M], 313 (100) [M-CH_3_], 73 (100).
Stearic Acid ^[b]^	356 (20) [M], 341 (90) [M-CH_3_], 327 (2) [M-CH_3_- CH_2_], 313 (50) [M-CH_3_-2xCH_2_].
Lactic Acid ^[b]^	219 (6) [M-CH_3_], 190 (14) [M-CO_2_], 147 (71) [M-Si(CH_3_)_3_-CH_3_], 133 (7), 117 (76) [M-Si(CH_3_)_3_-(CH_3_)_3_].
Pyruvic acid ^[b]^	160 (10) [M], 145 (7) [M-CH_3_], 88 (14) [M-Si(CH_3_)_3_], 71 (12) [M-Si(CH_3_)_3_-OH], 43 (100) [M-HSi(CH_3_)_3_- CO_2_].
Glucose ^[e]^	437 (5) [M-Si(CH_3_)_3_-2xCH_3_], 394 (4) [M-2xSi(CH_3_)_3_], 305 (5) [M-OSi(CH_3_)_3_-2xSi(CH_3_)_3_], 217 ^[^^g]^ (30), 204 ^[g]^ (100), 191 ^[g]^ (75).
Fructose ^[e]^	437 (5) [M-Si(CH_3_)_3_-2xCH_3_], 217 ^[g]^ (30), 204 ^[g]^ (100), 146 (75).
Glucitol ^[f]^	319 (60) [M], 297 (94) [M-CH_3_], 282 (30) [M-2xCH_3_], 267 (40) [M-3xCH_3_]; 217 ^[g]^ (90), 204 ^[g]^ (80); 147 (40).
Glycerol ^[d]^	293 (3) [M-CH_3_], 263 (2) [M-3xCH_3_], 218 (20) [M-OSi(CH_3_)_3_], 205 (60) [M-OSi(CH3)_3_-CH_3_], 191 (3) [M-OSi(CH_3_)_3_-2xCH_3_], 171 (4) [M-OSi(CH_3_)_3_-3xCH_3_].
Ethylene Glycol ^[c]^	191 (25) [M-CH_3_], 147 (100) [M-4xCH_3_], 133 (5) [M-Si(CH_3_)_3_], 103 (20) [M-Si(CH_3_)_3_-2xCH_3_].

[a] Mass spectroscopy was performed by using a GC-MS Varian 410 GC-320 MS. The peak abundance is reported in parenthesis [b] Product analyzed as the monosilyl derivative; [c] Product analyzed as the bis-silyl derivative; [d] Product analyzed as the tris-silyl derivative; [e] Product analysed as the penta-silyl derivative; [f] Product analysed as the hexa-silyl derivative; [g] Ions characteristic for EI/MS sugar degradation: *m/z* 217 [(CH_3_)_3_SiOCH=CH-CH=OSi(CH_3_)_3_]^+^, *m/z* 204 [(CH_3_)_3_SiOCH=CHOSi(CH_3_)_3_]^+•^, *m/z* 191 [(CH_3_)_3_SiOCH=OSi(CH_3_)_3_]^+^.

## Data Availability

Not applicable.

## Supplementary Materials

The following are available online at https://www.mdpi.com/article/10.3390/jof7100859/s1, Figure S1: Raman signal coverage, Figure S2: Raman peak position.
