# Supplementary material for: Fungal Biomarkers Stability in Mars Regolith Analogues after Simulated Space and Mars-like Conditions
Source: J Fungi (Basel). 2021 Oct 14;7(10):859. doi: 10.3390/jof7100859 (PMC8540304; doi:10.3390/jof7100859)
Supplement: Supplementary file 1 [file jof-07-00859-s001.zip › jof-1380693-supplementary.pdf]

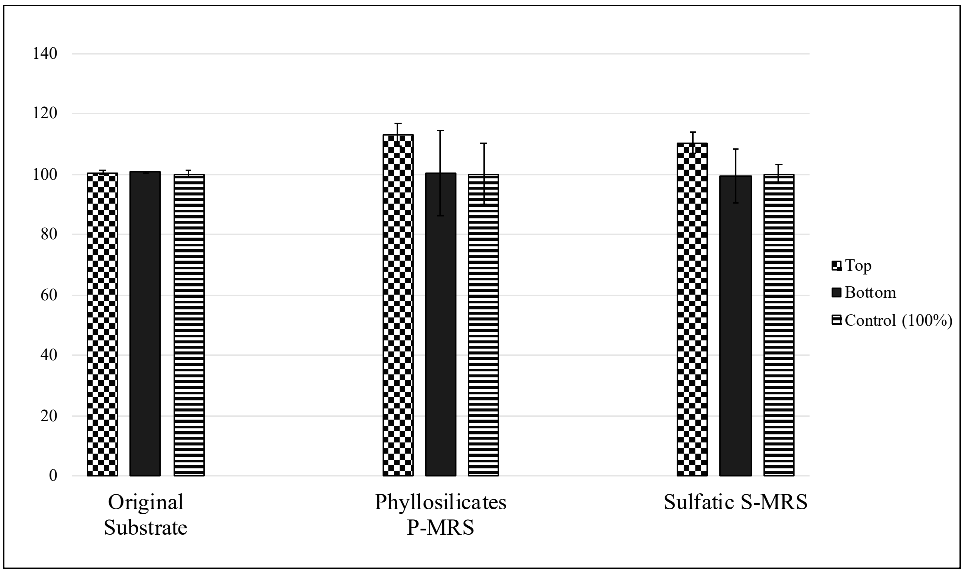

Figure S1. Signal coverage (%) calculated by applying a SNR mask superior to 4 for each exposure conditions (Top, Bottom, and Control) and substrata (OS, P-MRS, and S-MRS). Histogram shows no significant statistical difference between Top, Bottom and Control, that means no significant difference either between simulated space exposure and Mars-like conditions on spectra.

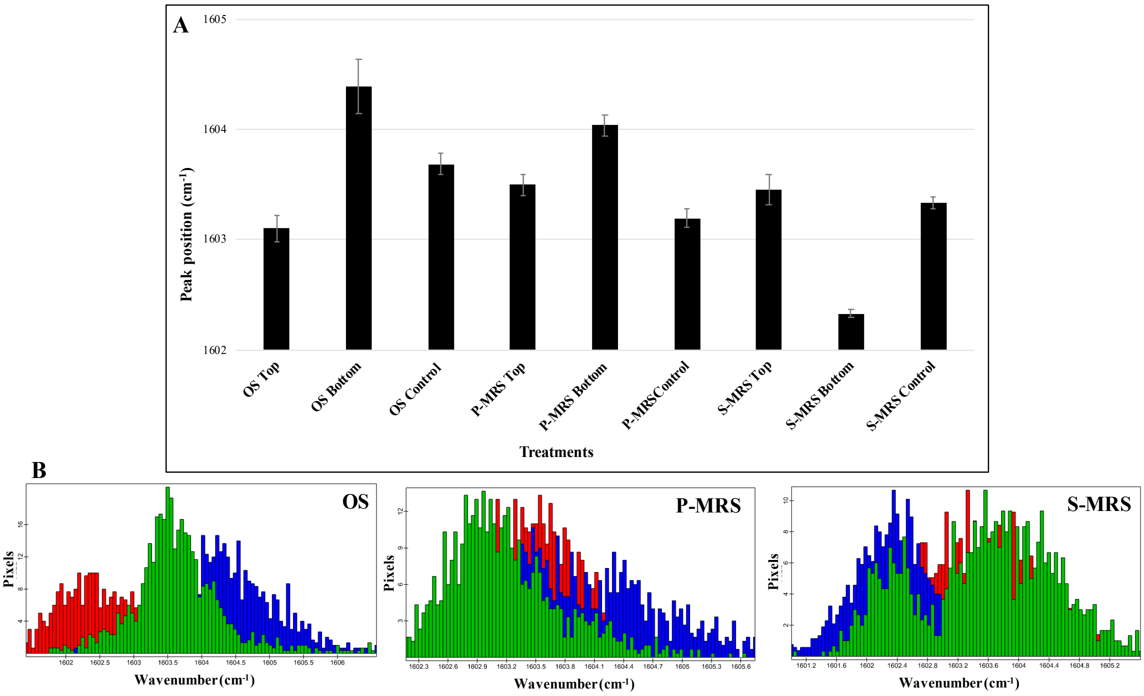

Figure S2. Confocal Raman Spectroscopy. **A**) Peak position for each exposure conditions (Top, Bottom, and Control) and substrata (Original Substrate: OS, Phyllosilicatic Mars Regolith Simulant: P-MRS, and Sulfatic Mars Regolith Simulant: S-MRS). Histogram shows no significant statistical difference between Top, Bottom and Control, that means no significant difference either between simulated space exposure and Mars-like conditions on melanin peaks. **B**) Distribution of the 1604 cm^{-1} melanin peak position is shown on the histogram for Top (red), Bottom (blue), and Control (green) for OS, P-MRS, and S-MRS samples.
